# Nth Slice Eraser: An Automated Algorithm to Ease Editing Workflow of Organ Contours Generated by Artificial Intelligence

**DOI:** 10.7759/cureus.73640

**Published:** 2024-11-13

**Authors:** Arjun Karnwal, Brittney Chau, Blake Chang, Marios P Tsotras, Colin Yeo, Arthur J Olch, Kenneth Wong

**Affiliations:** 1 Alfred E. Mann Department of Biomedical Engineering, University of Southern California, Los Angeles, USA; 2 Department of Radiation Oncology, University of Southern California Keck School of Medicine, Los Angeles, USA; 3 College of Humanities and Sciences, Stanford University, Los Angeles, USA; 4 Thomas Lord Department of Computer Science, University of Southern California, Los Angeles, USA; 5 Medical School, University of Southern California Keck School of Medicine, Los Angeles, USA

**Keywords:** deep learning artificial intelligence, eclipse, radiation oncology, radiation oncology training, radiation therapy contouring, treatment planning automation

## Abstract

The time-consuming process of manual contouring of healthy tissue and organs in radiation therapy has prompted the development of computational systems to aid and automate this process, such as artificial intelligence (AI) segmentation and interpolation algorithms. These algorithms are useful in saving time, however, they are not always accurate. Fixing such inaccuracies by editing contours is a manual, time-consuming process as no ‘undo’ feature currently exists in the most commonly used treatment planning system (TPS). In this short technical report, we present our script for automated de-interpolation to enable faster editing of contours to streamline the adoption of AI-assisted methodologies to enhance clinical workflow.

## Introduction

Modern radiation therapy planning begins with the contouring or segmentation of normal organs-at-risk (OAR) and target volumes. Contouring is a time-consuming task that is usually performed manually by radiation oncology professionals, including radiation oncologists, physicists, and dosimetrists [1]. Within the last decade, there has been an exponential increase in artificial intelligence (AI) for radiation planning, leading to new auto-contouring models that are assessed geometrically, qualitatively, and dosimetrically [2,3]. 

During manual segmentation, the standard practice is for a medical professional to contour non-adjacent CT slices while using a linear, shape-based, or machine-assisted interpolation algorithm to fill in the contours for intervening slices [4,5]. However, this manual approach tends to be time-consuming, an issue that has been addressed recently by the development of "auto-segmentation" algorithms [6]. The general procedure of auto-segmentation is to use an auto-contouring tool to generate the whole OAR contour before making manual adjustments to correct inaccuracies [7,8]. However, manually editing auto-contours may still be cumbersome, particularly when adjusting many adjacent slices at once. An approach that allows for the adjustment of important non-adjacent slices by enabling the erasure and re-interpolation of the gaps, could prove to be more time-efficient than manual editing; however, a de-interpolation (“eraser”) tool of AI-generated contours does not exist within the most commonly used treatment planning system (TPS). In this short technical report, we present our script for de-interpolation. 

## Technical report

Eclipse (Varian Medical Systems, Palo Alto, USA) is the most commonly used TPS in radiation oncology clinics worldwide. To automate de-interpolation of non-contiguous slices, a C#-based write-enabled script was developed to interact with the Eclipse TPS using the Eclipse Scripting Application Programming Interface (ESAPI). 

Depending on how many slices the user wishes to de-interpolate, the script can either clear every 1 in n (where n is an integer from 2 to 4) slices, or keep every 1 in n slices (clear every 2 out of 3 slices, or (n-1) out of n slices). 

First, after launching the script in Eclipse, a graphical user interface (GUI) appears. From here, the user selects which structure(s) they wish to de-interpolate. Next, they will select whether to ‘keep’ or ‘erase’ every 1 in n/interval slices. Selecting ‘keep’ will clear every 1 in n slices, and selecting ‘erase’ will clear every (n-1) out of n (3 out of 4, or 2 out of 3) slices. Finally, the user can select the interval or n of their choice. 

After all the settings are configured, the user clicks “De-Interpolate,” and the script execution begins. Since not all CT slices contain contours from the chosen structures, the script starts by iterating through every CT slice, searching for the first slice in which the structure to be edited has a contour. This first slice is taken as the starting layer.

**Figure 1:**
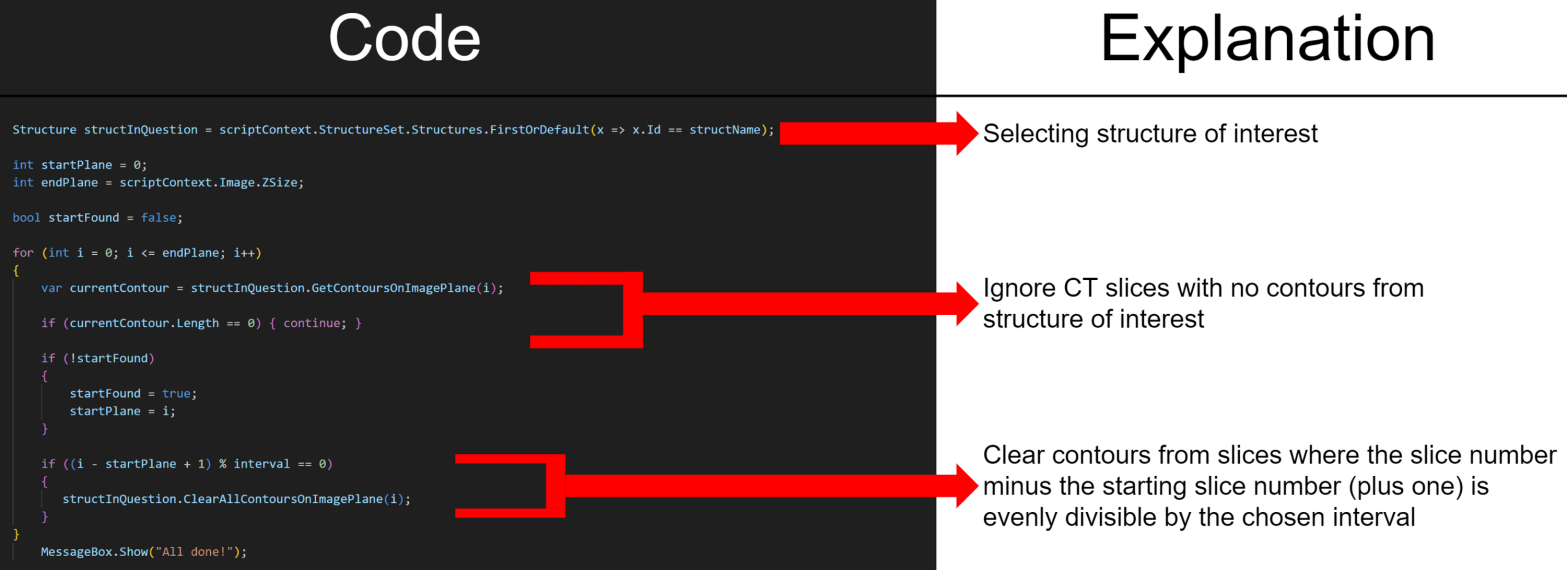
C# based script code for erasing every one in n slices

Next, the script iterates through all subsequent layers that contain a contour from the chosen structure(s). If the difference between the subsequent layer number and the starting layer number plus one is evenly divisible by the chosen interval (is a perfect/whole number multiple of), the subsequent layer is cleared of all contours from the chosen structure(s) (Figure 1).

For example, consider a structure that has contours on slices 10-20 of a 30-slice image set, in which the user wishes to erase every one in 3 slices (interval of 3). The script will start from slice 1, and look at all slices sequentially, ignoring slices 1-9 as they do not contain contours from the chosen structure. Once it reaches slice 10, it sets slice 10 as the starting layer and moves on to the next slice, slice 11. Since the difference between slices 11 and 10 (current slice and starting slice) plus one comes out to 2 (11-10 + 1), and since 2 is not evenly divisible by 3, this slice is skipped/ignored. The next slice, however, slice number 12, will be cleared of contours from the chosen structures. This is because the difference between the current slice (12) and the starting slice (10) plus one is 3 (12-10 +1), and 3 is evenly divisible by 3 (our desired interval). Likewise, slices 13 and 14 are ignored, since the difference between slice numbers and the starting slice plus one are 4 and 5, respectively, and neither of these are evenly divisible by 3. However, slice number 15 is cleared of contours from the chosen structure since the difference plus one here is 6, which is evenly divisible by 3. The process continues for all slices in the image set (until slice 30 in this example), and slices after slice 20 are all ignored since the structure no longer has contours after slice number 20. The process is repeated for all structures chosen by the user. This results in a robust methodology for clearing contours on every nth slice (See Figure 1). 

**Figure 2:**
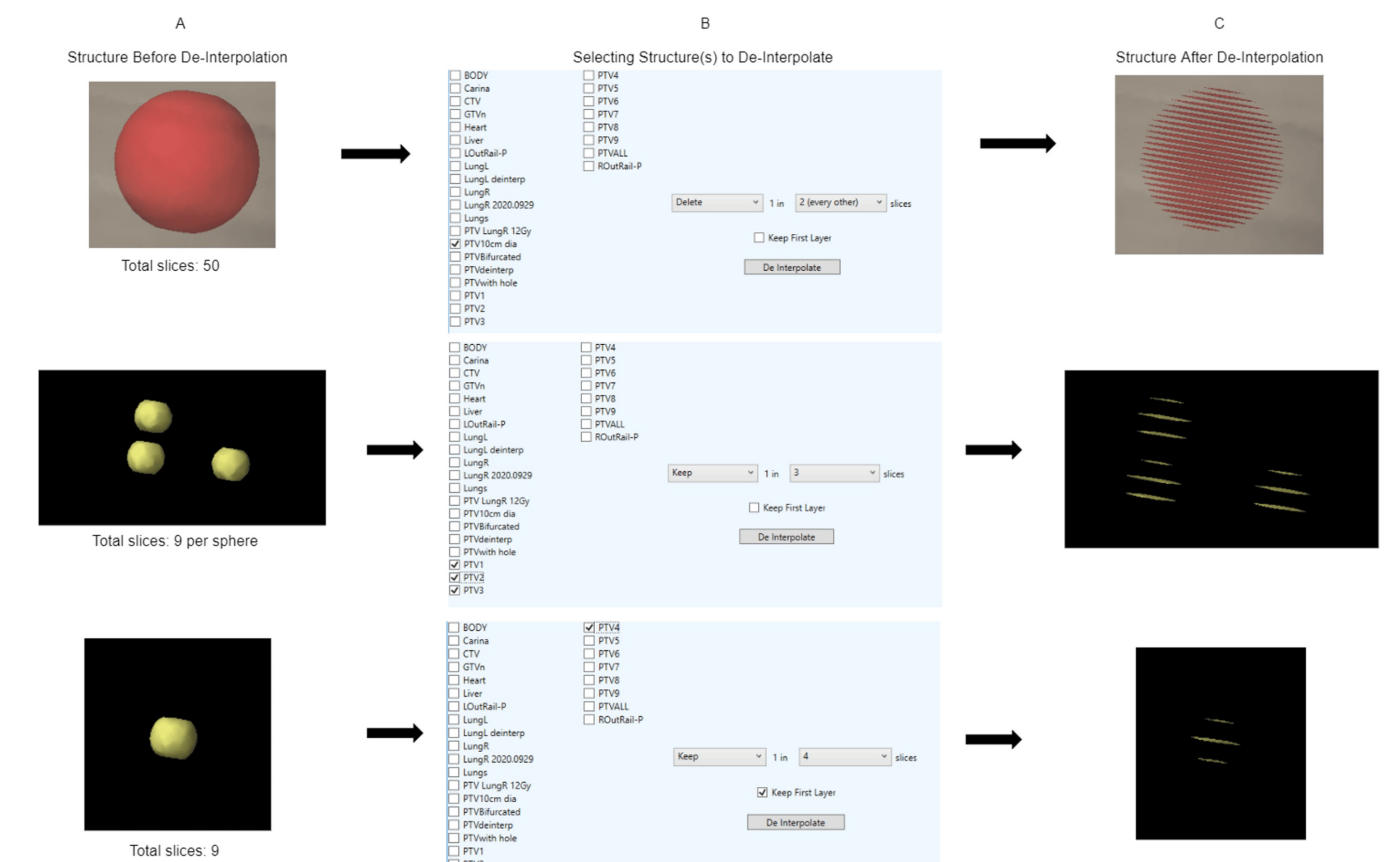
Script workflow and results Three examples of structures before de-interpolation (left). User interface with all options (middle). Structures after de-interpolation (right).

This workflow is the exact same for the ‘erase’ option, except for clearing on slices where the difference is evenly divisible by the interval/n, it clears wherever the slice difference is not evenly divisible by n. For the previous example, since we started on slice 10, and our interval was 3, slices 10 and 11 would be cleared, while slice 12 would remain untouched (clearing every two out of three slices). This is because the difference in slice numbers plus one for slices 10 and 11 are 1 and 2, respectively, and neither of these is evenly divisible by the interval, 3. Likewise, slices 13 and 14 would be cleared, while slice 15 would be ignored. The GUI also features a checkbox for whether the user would like to ignore the first slice of the structure or let it be cleared as a part of the script execution (keep the first slice, clear the next two, keep the fourth slice, clear the next two, etc or clear the first two, keep the third slice, etc). A sample of the user interface and the pipeline is shown in Figure 2.

**Figure 3:**
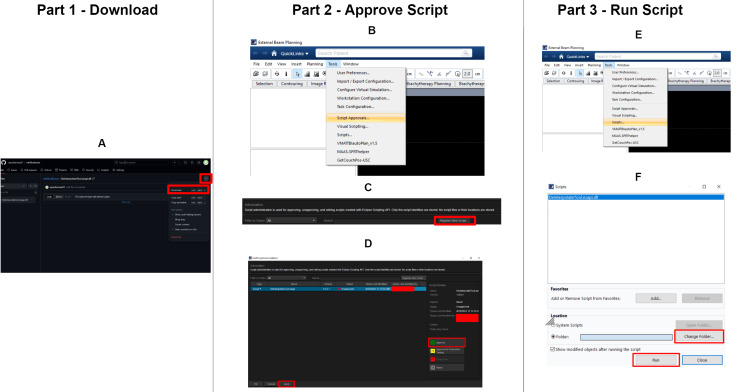
Script installation tutorial A. Download the script from https://github.com/arjunKarnwal27/nthSliceErasor/blob/main/DeInterpolateTool.esapi.dll B. In the Eclipse TPS, open the External Beam Planning interface. C. Approve the script by clicking “Script Approvals” → “Register New Script” → navigate to the location where the script is (was downloaded to) and open it. Click “Approve” on the right-hand side. D. In the External Beam Planning interface, select “Tools” and then “Scripts.” Navigate to where the script is located, and you should see one titled "DeInterpolate" (.esapi.dll) - select this one (or one with a similar name) and click “Run.” E. All set! Choose the structures you wish to de-interpolate, modify the interval as needed, and then select “De-Interpolate.” For help, email the lead author. TPS: treatment planning system

Like all other write-enabled ESAPI scripts, this program must be downloaded and approved to modify structures. The process for obtaining and using this software is outlined in Figure 3.

## Discussion

While this script is useful for AI-generated contours, it is also useful for rapid editing of contours generated by trainees, dosimetrists, and covering attendings. In many training institutions, attendings will instruct their trainees to not interpolate organ and target contours until after the attendings’ review. However, with this script, this practice will no longer be necessary and will be rendered outdated. This script will allow trainees to practice fully completing and editing their contours in these instances while attending supervision is improved by the ability to edit trainee contours effectively and quickly.

**Figure 4:**
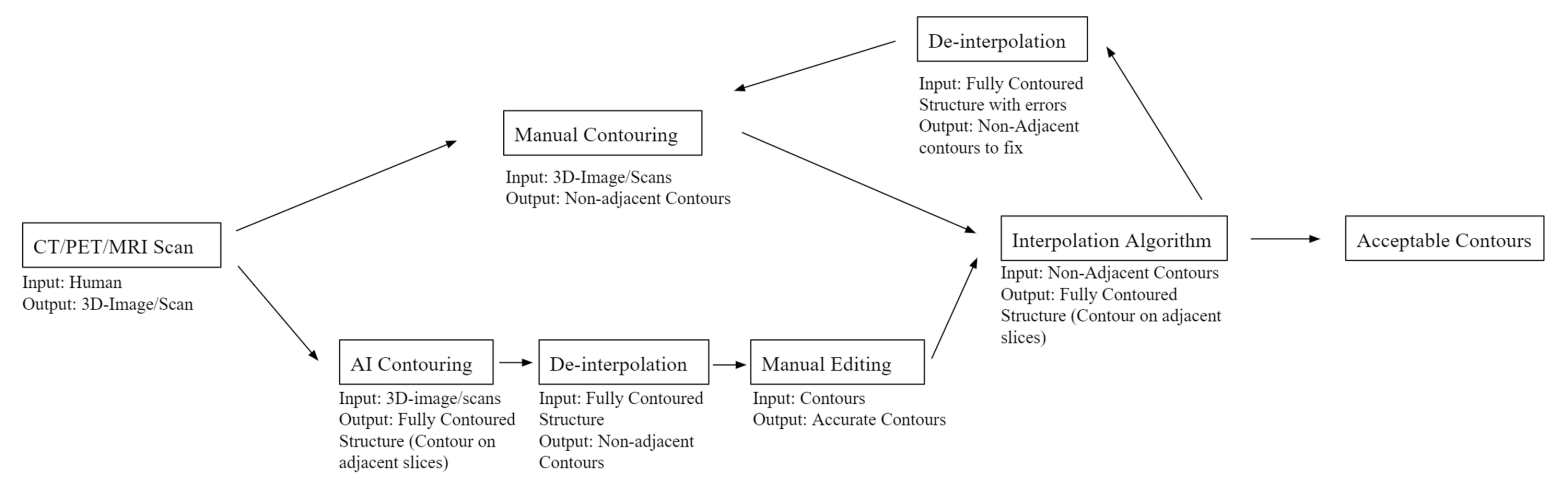
Contouring workflow with de-interpolation Author Flowchart Credit: Blake Chang

Furthermore, this script poses benefits beyond its ability to streamline the editing process for trainee contours; as the usage of auto-contouring software increases among radiation oncology professionals, this tool will ease the onus of current editing options of manually editing each auto-contoured slice, manually de-interpolating the structure, or simply erasing the auto-contoured structure and contouring it oneself (Figure 4). This may allow more time to review OARs and target volumes, for which there is a recognized variation in radiation oncology. Studies in various anatomical sites of disease, including pelvic, head and neck, and thoracic cancers have shown variations in contours, which can affect toxicities, local progression, and patient outcomes, even though several consensus statements and guidelines have been published [9,10,11]. This script can allow for more efficient use of auto-contouring software, which is another step towards standardizing contouring practices. 

Using linear, shape-based, and machine-assisted algorithms to interpolate contours is a well-established practice, whereas the “undo” or de-interpolate function is not provided. We provide a free, easy-to-use, robust approach to de-interpolate contours. 

Next steps

Future work will focus on enhancing customizability for the user, such as copying the structure and making edits on the copied structure (keeping the original structure as-is, in case the user wants to undo the de-interpolation), and allowing a start/stop layer (region of interest) selection. The latter would enable the user to limit the de-interpolation to a desired interval, in case they wish for the bottom half of the structure, for example, to remain untouched by the script. 

## Conclusions

This script potentially saves time when editing contours, whether generated by AI or manually by radiation oncology professionals. For AI-generated contours, congruence with manual contouring is high, though manual editing is necessary and expected when AI-generated contours are inaccurate. This script eases the manual editing process, thus facilitating the adoption of these AI-based tools to enhance workflow and potentially improve patient outcomes.
